# Molecular Prevalence of Selected Tick-Borne Pathogens in *Dermacentor reticulatus* Collected in a Natural Park in Italy

**DOI:** 10.3390/pathogens11080887

**Published:** 2022-08-08

**Authors:** Luca Villa, Sergio Aurelio Zanzani, Michele Mortarino, Alessia Libera Gazzonis, Emanuela Olivieri, Maria Teresa Manfredi

**Affiliations:** 1 Department of Veterinary Medicine and Animal Sciences, Università degli Studi di Milano, Via dell’Università, 6, 26900 Lodi, Italy; 2 Istituto Zooprofilattico Sperimentale della Lombardia e dell’Emilia Romagna, Strada Campeggi, 59/61, 27100 Pavia, Italy

**Keywords:** *Dermacentor reticulatus*, questing ticks, piroplasmida, *Babesia canis*, babesiosis, dog, spotted fever group, *Rickettsia*, TIBOLA, vector-borne diseases

## Abstract

*Dermacentor reticulatus* is one of the most important vectors of tick-borne pathogens (TBPs) in Europe causing diseases in animals and humans. A longitudinal study was planned, aimed to detect the molecular prevalence of tick-borne pathogens, i.e., *Babesia* spp. and the spotted fever group Rickettsiae, and its seasonal variation in *D. reticulatus* questing ticks to define the temporal infection risk. Ticks were collected monthly over a period of 15 months in a peri-urban park in Lombardy, Italy. DNA extraction and molecular analyses were performed. Statistical analysis was carried out. Out of 488, 53 (P = 10.9%) adult questing ticks were positive for *Babesia* DNA. A higher prevalence was revealed in male (32/241, P = 13.3%) than in female (21/247, P = 8.5%) ticks. Positive ticks were mostly collected in winter months (P = 13.3%) compared to early (P = 7.9) and late (P = 12.8) spring months. A similar percentage of positive ticks was evidenced in transects 1 and 3 (5.8% and 6.5%, respectively); instead, a significant higher prevalence was recorded in transect 2 (P = 16.0%). Obtained sequences confirmed a homology of 100% with *B. canis* sequences deposited in GenBank. No ticks tested positive for *Rickettsia* spp. DNA (0/488, P = 0%). The conspicuous circulation of *B. canis* infection in *D. reticulatus* adult questing ticks confirms their role in the epidemiology of canine babesiosis and requires preventive measures for dogs in this recreational area. Even if no tick was positive for the spotted fever group Rickettsia, its capacity as a vector of zoonotic pathogens should not be neglected.

## 1. Introduction

In the last few years, the changes in the distribution and abundance of tick species, due to both human factors and climate change, have caused an increase in the frequency of the vector-borne diseases of pets in Europe [1]. Indeed, ticks’ distribution is indicative of the risk of infection to receptive hosts, even if the presence of the potential vector does not directly implicate that of the transmitted pathogen [2].

*Dermacentor reticulatus* (Fabricius, 1794), the ornate dog tick or the marsh tick, is one of the most important vectors of tick-borne pathogens (TBPs) in Europe, causing diseases in animals and humans. Indeed, it represents, second only to *Ixodes ricinus*, the most common tick species in the region [3].

*D. reticulatus* is a Palearctic tick species with a highly focal and sporadic geographic range in Europe with a gap separating eastern and western macroregions [4,5]. In the last decades, its distribution has considerably expanded in some European regions [6]. Larvae and nymphs of *D. reticulatus* mainly feed on rodent species, particularly voles of the genus *Myodes* [7], whereas adults feed on large domestic and wild mammals, including ungulates, carnivores, equids, and pigs [8] but only occasionally bite humans [9]. In temperate Europe, adult ticks are generally active in spring with a peak in April; then their activity declines during summer months and they re-present in autumn with a usually smaller activity peak in September–October [10,11,12,13]. However, climatic conditions in different geographical areas can considerably vary the seasonal activity of *D. reticulatus* adults [14]. Regarding ecological aspects, the tick prefers wet and cold habitat types, such as fields, lands, pastures, forest paths, mixed or oak forests, or proximity to stagnant water, coasts, rivers and lakes [14]. The occurrence of *D. reticulatus* has also been observed in urban and suburban areas in the presence of natural hosts for adults, where dogs are common [15,16,17].

*D. reticulatus* is the proven vector of *Babesia canis*, one of the protozoan agents of canine babesiosis, a significant hemoparasitic disease of dogs, widespread in central and north-eastern Europe, which causing hemolytic anemia, splenomegaly, thrombocytopenia, and fever [18,19]. However, it can also transmit other pathogens of both veterinary importance, such as *B. caballi*, *Theileria equi* and *Anaplasma marginale*, and of public health relevance, such as *Rickettsia* spp. and some tick-borne encephalitis viruses [14].

As regards *B. canis*, the transovarial and transstadial transmissions of the protozoan in *D. reticulatus* ticks constitute the key routes enabling the maintenance of *B. canis* in tick populations [20]. The expansion in the geographical range of this tick species is clearly associated with the emergence of canine babesiosis in many European countries [6]. In Italy, *D. reticulatus* was only rarely reported in northern and central regions [21,22]. Moreover, only few data are available on *Babesia* spp. infecting dogs in Italy [23]. Recently, the circulation of *D. reticulatus* and the association of the tick with *B. canis* infection in dogs was reported in northern Italy [24].

Among the Gram-negative bacilli belonging to *Rickettsia* spp., *R. slovaca*, and *R. raoultii* are the two spotted fever Rickettsiae groups transmitted by *D. reticulatus*. These pathogens are recognized as the causative agents of tick-borne lymphadenopathy (TIBOLA), one of the most common tick-borne rickettsioses in Europe, clinically characterized by an eschar at the site of the tick attachment (on the scalp) surrounded by erythema and regional and painful lymphadenopathies [14,25,26].

The aim of this study was to expand knowledge on select pathogens potentially transmitted by *D. reticulatus* collected in a natural park in Italy and evaluate the health risk for humans and animal hosts. Therefore, a longitudinal study was planned aimed to detect the molecular prevalence of tick-borne pathogens, i.e., *Babesia* spp. and spotted fever group Rickettsia, and its seasonal variation in *D. reticulatus* questing ticks to define the temporal infection risk. Indeed, the circulation of pathogens in questing ticks offers an insight into the risk of disease transmission and potential implicated vectors.

## 2. Results

A total of 488 *D. reticulatus* questing ticks were processed. All of them were adult ticks; no immature stages were collected in any sampling session. The overall sampling included 241 female and 247 male specimens. The number of *D. reticulatus* collected varied over the months of sampling: questing ticks were collected from April to June 2015 and from January to June 2016. No ticks were found from July to December 2015. The highest ticks’ numbers were recorded in 2016, particularly in March and April (Table 1) [17].

Overall, 58 specimens were positive for piroplasmid DNA, resulting in a molecular prevalence (P) of 11.9%. The amplification of 408-bp fragments of *Babesia* spp. was successful for 53 DNA samples, with a prevalence of 10.9%. The prevalence was higher in male (32/241, P = 13.3%) than in female (21/247, P = 8.5%) ticks. *Babesia* spp. DNA positive ticks were mostly collected in March (*n* = 28) and April (*n* = 17); however, positivity also occurred in February (*n* = 2) and May (*n* = 5). A similar percentage of positive ticks was evidenced in transects 1 and 3 (11/190, P = 5.8% and 4/61, P = 6.5%, respectively); instead, a higher prevalence was recorded in transect 2 (38/237, P = 16.0%). Molecular prevalence results of *Babesia* infection in *D. reticulatus* adult questing ticks sorted by sex and year and month of sampling are summarized in Table 1.

Out of 53 obtained *Babesia* spp. 18S rDNA amplicons, only 23 were successfully sequenced. BLASTn analysis confirmed a homology of 99.72% and 100% query cover with *B. canis* 18S rDNA partial sequences obtained from blood samples of Hungarian dogs showing clinical signs of babesiosis (GenBank accession numbers AY611729-AY611733) [27]. Since no intraspecific nucleotide variations were detected between any of the obtained *B. canis* sequences, one representative sequence was submitted to GenBank under accession number ON909757.

No ticks tested positive for *Rickettsia* spp. DNA (0/488, P = 0%) through real time PCR.

Statistical analysis did not evidence any difference in *Babesia* molecular prevalence considering ticks’ sex and season of collection (Table 2). Instead, a significant statistical association was revealed for the collection site, with ticks collected in transect 2 at increased risk of protozoa infection, the risk being 2.530 times higher than that of ticks collected in the other two transects (Table 3).

## 3. Discussion

The circulation of *B. canis* in *D. reticulatus* in a peri-urban park in Italy was confirmed. In a previous survey [24], a percentage equal to 12.3% of dogs showed positivity to *Babesia* by serological and/or molecular analyses. Moreover, molecular analysis on both the blood of dogs and ticks showed the nucleotide identity of *B. canis*, indicating the circulation of the same strain between hosts and vectors, suggesting that, in the surveyed area, this tick species seems most likely associated with dogs.

In the present study, a molecular prevalence of *Babesia* spp. of 10.9% in *D. reticulatus* adult questing ticks is reported. In particular, a higher percentage, even if not significant, of male than female specimens were found to be carriers of *Babesia* DNA. Since the feeding and the engorgement of male ticks is much less obvious and visible, and indeed male ticks are usually categorized as slightly engorged [28], it was suggested that these characteristics could make them harder to detect by pet owners, increasing the risk of parasite transmission. Aside from the ability of male *D. reticulatus* to transmit *B. canis* to dogs, an early transmission of the protozoa within 8 h of tick infestation in the case of interrupted-feeding behavior was also recently demonstrated [29]. In fact, in natural conditions, male ticks can detach spontaneously from their host and re-attach, as evidenced for *D. andersoni* [30]. On the other hand, it should be considered that this piroplasmid is able to invade the ovaries of female ticks and be transmitted transovarially to the next generation of larvae. Even if the efficiency of transovarial transmission in natural conditions requires further field research [20], together with transstadial transmission, these features enable *D. reticulatus* populations to function as a reservoir, allowing the maintenance of *B. canis* locally for several tick generations even without a vertebrate host [14].

Most *Babesia* positive ticks were collected in March and April, corresponding to the early spring single peak of activity of *D. reticulatus* [17]; however, some positive samples, both male and female, were also detected in late spring. Interestingly, only two positive male ticks were evidenced in February, implicating a potential risk for parasite transmission also in late winter. The seasonal difference in the activity of *D. reticulatus* males and females due to temperature and humidity changes could be associated with the morphological and physiological traits for the maintenance of favorable water balance [31].

The occurrence of *Babesia* DNA in questing ticks was similar in transects 1 and 3, but a higher prevalence was revealed in *D. reticulatus* from transect 2, presumably influenced by habitat type and host availability. Indeed, even if the presence of both dogs and humans was recorded in all the transects, transect 2 was composed mainly of bushes with sparse trees, whereas transects 1 and 3 were mainly composed by mixed forest with the dominance of oak forest. Furthermore, in the park the presence of several species of Arvicolines, known to be the preferred hosts for *D. reticulatus* immature stages, were noted. Even if specific data on micromammals population density are not available for the park, some Arvicolines species hosted here have a preferred habitat similar to that of transect 2. Then, the overall environmental features of the sampling sites may influence both the frequency and the possible contacts between hosts, vectors, and pathogens.

In recent years, canine babesiosis is considered an emerging infectious disease in dogs in Europe. Among others, the abundance of tick vectors, the percentage of infected ticks, and the *Babesia* species involved are the factors implicated in the emergence of the disease in European countries [32]. As previously mentioned, *Babesia* spp. are naturally transmitted only by ticks, and in the case of canine babesiosis, a strong association between the *Babesia* species and the tick species is recognized. In Europe, canine babesiosis is mostly caused by *B. canis* in association with *D. reticulatus* ticks [33]. Therefore, since the prevalence of babesiosis depends on the presence of the tick vector in the environment [32], the wide geographic distribution of *B. canis* is in line with that of *D. reticulatus* [14].

In Europe, clinical cases of canine babesiosis caused by *B. canis* were reported in Albania [34], Austria [35], Belgium and the Netherlands [36,37], Croatia [38], France [39], Germany [40], Hungary [27], Italy [41], Norway [42], Poland [43], Portugal [44], Slovenia [45], Spain [46], and Switzerland [47]. Based on the molecular screening of field-collected ticks, the prevalence of *B. canis* in adult *D. reticulatus* ticks varies in European countries from 0.3% to 20.8% [29,38,48,49,50,51,52,53,54,55]. In a recent systematic review and meta-analysis on the global distribution of *Babesia* spp. in questing ticks, the estimated pooled prevalence of *Babesia* spp. in *D. reticulatus* was 2.1%; in particular, the tick was associated with six different *Babesia* species, with *B. canis* being the most prevalent. Indeed, in most of the considered studies by the authors, *B. canis* DNA was reported in *D. reticulatus* ticks [56]. According to Hornok et al. [54], the prevalence of the infected ticks was higher in February and March even if, in respect to the present study, these authors found infected ticks also in December, and the prevalence values in Hungary were also higher (43.5%) than those registered in *D. reticulatus* from the studied Italian peri-urban park. The finding of *Babesia* DNA in *D. reticulatus* seems suggest a seasonal pattern of *B. canis* infected *D. reticulatus*, coinciding with the seasonal occurrence of the clinical cases of babesiosis in dogs registered in the study area and also in Central Europe [47,54,57].

Regarding the natural cycle of *B. canis*, no known wildlife reservoir host has been yet identified, suggesting the probable reservoir role of dogs in the life cycle, and also the evolutionary origin of the pathogen in domesticated dogs. This fact could also explain the recent geographical spread of canine babesiosis with the introduction of local *D. reticulatus* in the new area from infected dogs and the short-term maintenance by transovarial and transstadial transmission in infected ticks [14]. Moreover, the protozoan was also found in other carnivorous hosts, such as the Eurasian golden jackal, the grey wolf, and the red fox; therefore, it was hypothesized that these canids may play a role in maintaining *B. canis* foci in areas where they are present together with domestic dogs, as evidenced in the Czech Republic [57].

Finally, in the present study, the circulation of spotted fever group rickettsia in *D. reticulatus* was not evidenced. Variable high prevalence values of *R. raoultii* were observed in questing *D. reticulatus* adults in other European countries; instead, *R. slovaca* is usually not detected or only with lower prevalence in this species, since it occurs more often in *D. marginatus* ticks [14]. A recent study conducted in northwestern Italy reported a prevalence of 33.3% of *R. slovaca* in *D. reticulatus* ticks collected on wild boars from a peri-urban area nearby Turin city [58]. However, other Italian studies also demonstrated the presence of the spotted fever group rickettsia in *D. marginatus* ticks [59,60,61]. The absence of *Rickettsia* spp. in *D. reticulatus* ticks from our study may be due to the characteristics of the study area: indeed, it is a relatively small, close, peri-urban park, with limited animal biodiversity; this differs to extended natural parks where the presence of suitable sylvatic hosts, e.g., ungulates, may favor not only the cycle of the tick but also the maintenance of the *Rickettsia* infection. To date, little is known of the enzootic cycles of *R. raoultii* and *R. slovaca*. It is reported that ticks could be main reservoir of the spotted fever group *Rickettsia* due to co-feeding transmission between ticks from the same generation in combination with transstadial and transovarial transmission. However, vertebrate hosts may also be necessary to maintain and perpetuate certain rickettsial agents in nature [62], even if it is still unclear which vertebrate hosts are involved in the amplification of *Rickettsia*-infected ticks [14].

The use of molecular-based techniques for diagnosing tick-borne pathogens has been widely adopted due to its high sensitivity and specificity. In this study, real-time PCR was used as screening due to higher sensitivity when compared to conventional PCR. Moreover, even if the sequencing only yielded an interpretable result for a part of the samples, this result is in agreement with other studies and could be probably due to a low DNA content [54,57]. Indeed, Hornok et al. [54] and Daněk et al. [57] produced similar results to those reported in the present study detecting the DNA of *Babesia* spp. in 34 out of 413 (8.2%) and in 74 out of 2497 (3.0%) adult ticks, respectively.

As previously evidenced [17], the conditions of this peri-urban park are favorable for the establishment of foci of *D. reticulatus*, both for the type of habitat and the climatic conditions, and also for the availability of suitable hosts. However, the dynamics of the investigated pathogens in *D. reticulatus* adult ticks resulted in differences in the study area. Indeed, the circulation of *B. canis*, already reported by [24], was confirmed, probably linked to the reservoir role of dogs, regularly present in the park, and the maintenance in ticks for transovarial and transstadial transmission. Regarding other canids that could act as an animal reservoir of *B. canis*, these parasites were rarely recorded in free-living canids, red foxes and grey wolves, and it should also be considered that the occurrence of the red fox was only rarely reported in the surveyed park [57]. On the other hand, the characteristics of the park may be not suitable for the circulation of the spotted fever group *Rickettsia*, considering the lack of appropriate vertebrate species for rickettsial agents.

## 4. Materials and Methods

### 4.1. Sample Collection

The majority of analyzed ticks were collected in a previous sampling aimed to study the seasonal dynamics of *D. reticulatus* performed during the years 2015 and 2016 in a peri-urban park in Italy [17]. Additional samples collected in June 2016 were also considered. The study area was the Groane Regional Park, located in the peri-urban area of Milan and Monza Brianza provinces (Lombardy). This environment is characterized by mixed forest with several rivers and ponds, interspersed with roads, housing, and agricultural areas. The presence of suitable hosts for ticks (e.g., dogs, horses, cattle, wild ungulates, and voles) is documented in the park [63].

Ticks were collected along five permanent transects [17], but only those collected in transect 1, 2 and 3 were included in this study; indeed, only one tick was collected in each of transects 4 and 5, respectively. Transects 1 and 2 were forest paths for pedestrians and bikers with picnic and recreational sites, close to human-inhabited and industrial areas; transect 3 was a forest path exclusively used for dog training. A different type of vegetation was depicted: transects 1 and 3 were characterized by mixed forest with the dominance of oak forest, whereas transect 2 was mainly composed of bushes with sparse trees; in all transects there were ponds as water reservoirs. Considering the host availability for adult ticks, in all the transects a high frequency of both dogs and humans was reported. Ticks were collected over a period of 15 months from April 2015 to June 2016, using both dragging and flagging techniques on leaf litter and the vegetation. Ticks were separated by stage/gender and morphologically identified at the species level by taxonomic keys [64,65,66] and categorized by site of collection and date. Ticks were then preserved in 70% ethanol at room temperature from collection time until processing for DNA extraction and molecular analyses.

### 4.2. Molecular Analyses

Prior to DNA extraction, ticks were mechanically crushed in a lysis buffer, according to Halos et al. [67]. Then, obtained samples were processed to extract genomic DNA using a commercial kit (NucleoSpin^®^ Tissue, Macherey-Nagel, Düren, Germany), following the manufacturer’s instructions. Extracted DNA samples were quantified using a spectrophotometer (Nanodrop ND 1000, Thermo Scientific, Wilmington, DE, USA) and the quality was assessed by amplifying a mitochondrial gene coding 16S rDNA [4]. DNA samples were stored at −20 °C until the time of analysis.

DNA samples were initially subjected to a screening real time PCR for piroplasmids with primers targeting the 18S rRNA, as previously described [68]. The reactions were performed in a final volume of 20 μL, containing the PowerUp™ SYBR^®^ Green Master Mix (Thermo Fisher Scientific, Life Technologies, Monza, Italy), 2 × 0.5 μM of each primer (forward primer 5′-GACGATCAGATACCGTCGTAGTCC-3′ and reverse primer 5′-CAGAACCCAAAGACTTTGATTTCTCTC-3′), and 3 μL of DNA samples (approximately 250–500 ng of genomic DNA). Positive and negative (non-template) controls were included in each run; the positive control consisted of genomic DNA of *B. canis* from the blood of infected dogs [24]. Amplification and melting analysis were performed in a QuantStudio™ 3 Real-Time PCR System (Applied Biosystems™ LSA28137, Waltham, MA, USA) equipped with a QuantStudio™ 3 software system (Thermo Fisher Scientific, Life Technologies, Monza, Italy), with the following cycling profile: incubation at 50 °C for 2 min, denaturation at 95 °C for 2 min, amplification for 40 cycles at 95 °C for 15 s, 60 °C for 60 s, and a final melting analysis step. The melting program, comprising temperature increases from 60 °C to 95 °C at intervals of 0.15 °C/s, was performed at the end of each cycle. Each sample was analyzed in duplicates, and the mean cycle threshold (Ct) and melting temperature (Tm) values were recorded. A sample was defined as positive when there was (i) a detectable amplification curve, (ii) a Ct value below 35, and (iii) a Tm value of ±0.5 °C vs. Tm value of positive control.

On samples that were positive for piroplasmid DNA, a conventional PCR for *Babesia* spp. and subsequent sequencing was carried out, using primers and protocols previously described [69]. Real-time positive DNA samples were analyzed using conventional PCR targeting a region of 408 bp of the 18S rRNA gene. The reactions were performed in a final volume of 50 μL, containing the DreamTaq Green PCR Master Mix (Thermo Fisher Scientific, Life Technologies, Monza, Italy), 2 × 0.5 μM of each primer (PIRO-A 5′-AATACCCAATCCTGACACAGGG-3′, PIRO-B 5′-TTAAATACGAATGCCCCCAAC-3′), and 5 μL of DNA samples (approximately 250–500 ng of genomic DNA). The same positive and negative (non-template) controls described above were inserted in each run. The PCR reactions were performed in a thermal cycler (Applied Biosystems SimpliAmp Thermal Cycler, Waltham, MA, USA). The reaction was performed with an initial denaturation step of 95 °C for 3 min, followed by 40 cycles of denaturation (30 s at 95 °C), annealing (30 s at 58 °C) and extension (1 min at 72 °C), and a final extension step at 72 °C for 10 min. PCR products were run on 1.5% agarose gel containing 0.05% ethidium bromide in TBE buffer electrophoresis and visualized under UV light on a transilluminator using a 100 bp DNA ladder (GeneRuler, Thermo Fisher Scientific, Life Technologies, Monza, Italy) as a size standard. Bands of the expected size were excised from agarose gel, purified with a commercial kit (NucleoSpin^®^ Gel and PCR Clean-up, Macherey-Nagel, Düren, Germany) following the manufacturer’s instructions, and finally sent for bidirectional sequencing to a commercial service (Eurofins MWG Operon, Ebersberg, Germany). Electropherograms were checked, and consensus sequences were manually assembled. Sequences were compared to nucleotide sequences available in the GenBank using BLASTn (https://blast.ncbi.nlm.nih.gov/, accessed on 29 July 2022) and then aligned with sequences available in GenBank using the Mega6 software [70].

For the detection of *Rickettsia* spp., DNA samples were initially subjected to a screening real time PCR with primers targeting the ITS2 region, as previously described [71].

Following real time PCR positivity, samples underwent conventional PCR using primers CS-78F and CS-323R targeting a partial sequence (401 bp) of the gene citrate synthase (gltA), present in all species of *Rickettsia* [72]. Then, in cases of positivity, samples were subjected to a second PCR using the primers Rr190.70F and Rr190.701R targeting a fragment of the outer membrane protein A (ompA) gene (632 bp) [73], present only in spotted fever group (SFG) Rickettsia. The protocols of molecular analyses were performed according to Sgroi et al. [59]. Positive and negative controls (non-template) were included in each run; the positive control consisted of genomic DNA of *R. monancesis*, *R. raoultii*, and *R. slovaca* [59]. The same procedures and equipment as described for *Babesia* spp. were used.

### 4.3. Statistical Analysis

Only ticks collected in 2016 were considered for statistical analysis since a low number of ticks in one single sampling site was collected in 2015. Statistical analysis was only performed for *Babesia* spp. data since no tick was positive for *Rickettsia* spp.

A generalized linear model (GLM) was used to assess the influence of tick sex, season, and sampling site on tick positivity for *Babesia* spp. DNA. The following variables, i.e., sex (males and females), season (winter: January, February, and March; early spring: April; late spring: May and June) and sampling sites (transects 1, 2, and 3), were entered in the model as dependent variables. The binary outcome (negativity/positivity to *Babesia* spp.) based on conventional PCR results was used as the independent variable. The models were developed through a backward selection procedure (significance level to remove variables from the model = 0.05), based on Akaike information criterion (AIC) values. Statistical analysis was performed using SPSS software (Statistical Package for Social Science, IBM SPSS Statistics for Windows, v25.0., Chicago, IL, USA).

## 5. Conclusions

This study evidenced a conspicuous circulation of *B. canis* infection in *D. reticulatus* adult questing ticks and confirmed their role in the epidemiology of canine babesiosis. The detection of *B. canis* in questing *D. reticulatus* requires preventive measures for dogs in this frequented recreational area. Moreover, the risk of acquiring the disease for owned dogs attending the peri-urban park is considerable in spring months. Considering the efficient transstadial and transovarial transmission of *Babesia* spp. in ticks, tick prophylaxis is strictly required and should cover the entire period during in which *D. reticulatus* is active in order to prevent canine babesiosis. As previously evidenced [19], the application of molecular methods in eco-epidemiological studies may help to identify specific interactions between pathogens and tick species and the actual health hazard constituted by *Babesia* spp. and tick species in certain locations. Moreover, even if no tick was positive for the spotted fever group Rickettsia and *D. reticulatus* only seldom bites humans, their capacity as vectors of zoonotic pathogens should not be neglected.

## Figures and Tables

**Table 1:** Molecular prevalence of *Babesia* spp. in *Dermacentor reticulatus* adult questing ticks in the investigated peri-urban park in Italy sorted by year and month of sampling.

Year	Month	Ticks					
		Overall		Males		Females	
		Prevalence (*n* Positive/Examined)	95% CI	Prevalence (*n* Positive/Examined)	95% CI	Prevalence (*n* Positive/Examined)	95% CI
**2015**	**April**	33.3% (1/3)	0.84–90.57	33.3% (1/3)	0.84–90.57	0 (0/0)	0
	**May**	0 (0/6)	0	0 (0/4)	0	0 (0/2)	0
	**June**	0 (0/1)	0	0 (0/1)	0	0 (0/0)	0
	**July**	0 (0/0)	0	0 (0/0)	0	0 (0/0)	0
	**August**	0 (0/0)	0	0 (0/0)	0	0 (0/0)	0
	**September**	0 (0/0)	0	0 (0/0)	0	0 (0/0)	0
	**October**	0 (0/0)	0	0 (0/0)	0	0 (0/0)	0
	**November**	0 (0/0)	0	0 (0/0)	0	0 (0/0)	0
	**December**	0 (0/0)	0	0 (0/0)	0	0 (0/0)	0
**2016**	**January**	0 (0/1)	0	0 (0/1)	0	0 (0/0)	0
	**February**	16.7% (2/12)	2.09–48.41	25% (2/8)	3.19–65.09	0 (0/4)	0
	**March**	13.2% (28/213)	8.92–18.44	14.7% (17/116)	8.78–22.42	11.3% (11/97)	5.80–19.39
	**April**	7.9% (17/213)	4.72–12.47	9.9% (9/91)	4.62–17.95	6.6% (8/122)	2.87–12.51
	**May**	13.5% (5/37)	4.54–28.77	17.6% (3/17)	3.80–43.43	10% (2/20)	1.24–31.70
	**June**	0 (0/2)	0	0 (0/0)	0	0 (0/2)	0
**Total**		10.9% (53/488)	8.24–13.96	13.3% (32/241)	9.26–18.22	8.5% (21/247)	5.34–12.70

**Table 2:** Potential risk factors for *Babesia* spp. positivity in *Dermacentor reticulatus* by univariate analysis.

Variable	Category	Ticks *n* Positive/Tested	Prevalence	OR (95% CI)	*p*-Value
**Sex**	**Female**	21/245	8.6%	0.658 (0.376–1.152)	0.143
	**Male** (reference)	31/233	13.3%	1	
**Season**	**Winter**	30/226	13.3%	1.122 (0.408–3.081)	0.824
	**Early spring**	17/213	7.9%	0.705 (0.248–2.005)	0.512
	**Late spring** (reference)	5/39	12.8%	1	
**Sampling sites**	**Transect 1**	10/180	5.5%	1.147 (0.392–3.360)	0.803
	**Transect 2**	38/237	16.0%	2.530 (1.307–4.899)	**0.006**
	**Transect 3** (reference)	4/61	6.5%	1	

**Table 3:** Potential risk factors for *Babesia* spp. positivity in *Dermacentor reticulatus* by multivariate analysis.

Variable	Category	Ticks *n* Positive/Tested	Prevalence	OR (95% CI)	*p*-Value
**Sampling sites**	**Transect 1**	10/180	5.5%	1.147 (0.392–3.360)	0.803
	**Transect 2**	38/237	16%	2.530 (1.307–4.899)	**0.006**
	**Transect 3** (reference)	4/61	6.5%	1	

## Data Availability

The datasets used and/or analyzed during the current study are available from the corresponding author on reasonable request.
